# Upregulation of *Yy1* Suppresses Dilated Cardiomyopathy caused by *Ttn* insufficiency

**DOI:** 10.1038/s41598-019-52796-0

**Published:** 2019-11-08

**Authors:** Dan Liao, Weiming Chen, Chia Yee Tan, Jing Xuan Wong, Pui Shi Chan, Lek Wen Tan, Roger Foo, Jianming Jiang

**Affiliations:** 1 Department of Biochemistry, Yong Loo Lin School of Medicine, National University of Singapore, Singapore, 117597 Singapore; 2 Cardiovascular Research Institute, National University Health System, Centre for Translational Medicine, Singapore, 117599 Singapore; 3 Genome Institute of Singapore, A*STAR, Singapore, 138672 Singapore

**Keywords:** Cardiac hypertrophy, Molecular medicine

## Abstract

Truncating variants in *TTN* (TTNtv), coding for the largest structural protein in the sarcomere, contribute to the largest portion of familial and ambulatory dilated cardiomyopathy (DCM). *TTN* haploinsufficiency caused by TTNtv is suggested as the disease mechanism. However, it is unclear whether *TTN* insufficiency causes DCM. Moreover, it is unknown whether modulation of downstream pathways serves as a therapeutic strategy for DCM caused by *TTN* insufficiency. Here, we show that reduction of cardiac *Ttn* expression by adeno-associated virus mediated shRNA (*Ttn* shRNA) generated DCM in mouse, demonstrating impaired cardiac performance, enlarged left ventricle (LV) and reduced LV wall thickness. A screen of 10 dysregulated and selected genes identified that Yin Yang 1 (*Yy1*) significantly suppressed DCM caused by *Ttn* shRNA. Gene profiling by RNAseq showed *Yy1* modulated cell growth related genes. *Ttn* insufficiency activated cardiomyocyte cell cycle reentry by upregulating of *Ccnd1* and *Ccnd2*. Cardiomyocytes activated by *Ttn* insufficiency did not advance to S phase by EdU incorporation assay. *Yy1* promoted cardiomyocyte cell cycle by further enhancing *Ccnd1* and *Ccnd2* and increasing DNA replication without undergoing cell division. Importantly, upregulation of *Ccnd1* and *Ccnd2* suppressed DCM caused by *Ttn* insufficiency. Our findings demonstrate that DCM caused by *Ttn* insufficiency can be treated by therapeutically promoting cardiac cell cycle.

## Introduction

Dilated cardiomyopathy (DCM) occurs as many as 1 in 250 people^1^. There are currently no approved therapeutic products indicated for DCM treatment. Typical treatments are those indicated for broader cardiovascular disease. As the disease progresses, patients have limited treatment options, such as surgical or other invasive interventions and heart transplant^2^. DCM will result in heart failure with reduced ejection fraction (EF), usually without prior ischemic. The walls of the left ventricle are thin and over-expanded, leading to improper contraction and insufficient blood being pumped by the dilated heart. DCM results from a variety of external factors, such as viral infection, alcohol abuse, exposure to cardiotoxic medications and pregnancy, as well as from genetic variants in a number of causal genes including *TTN*, *LMNA*, *ACTC1*, *MHY7* and *PLN*^3–9^. Titin (coded by *TTN*) plays an important role in the contraction and relaxation of cardiac muscles by connecting Z-disc to the M-line in the sarcomere. *TTN* truncating variants (TTNtv) contribute up to 15% ambulatory DCM and 25% end-stage or familial DCM^3,8,9^. In DCM, TTNtv are significantly enriched most in A band as well as other regions including I-band, Z-disc or M-line with variable position-related odds ratios^3,10^. *TTN* haploinsufficiency caused by TTNtv is emerging as the potential disease mechanism. Rat models with TTNtv in Z-disc and A band did not result in change in titin protein levels and obvious cardiac performance under normal physiological conditions. It was not known whether *Ttn* insufficiency causes DCM in mouse.

Mammalian cardiomyocytes exit their cell cycle shortly after birth, preventing heart repair through cardiac regeneration^11^. Cell cycle reactivation is observed in a limited cardiomyocytes under physiological conditions^12,13^. As an emerging strategy for cardiac therapeutic regeneration, we and others showed that enhancing this process by removing cell cycle brakes or augmenting accelerators is beneficial for heart recovery from heart failure models caused by ischemia or pressure overload^14–18^. We previously observed that cardiomyocytes undergo an extra round of cell cycle in *Mybpc3* deficient mice, suggesting cell cycle reactivation could compensate sarcomere insufficiency^19^. It was not known whether *Ttn* insufficiency could induce cardiac cell cycle reactivation. Moreover, it was unknown whether enhancing this process could be a therapeutic strategy for DCM caused by *Ttn* insufficiency. Here, we address these major gaps and identify therapeutic candidates that are vital for advancing potential hits into a therapeutic approach for DCM.

## Results

### ***Ttn*** shRNA induces DCM in mice

**Figure 1:**
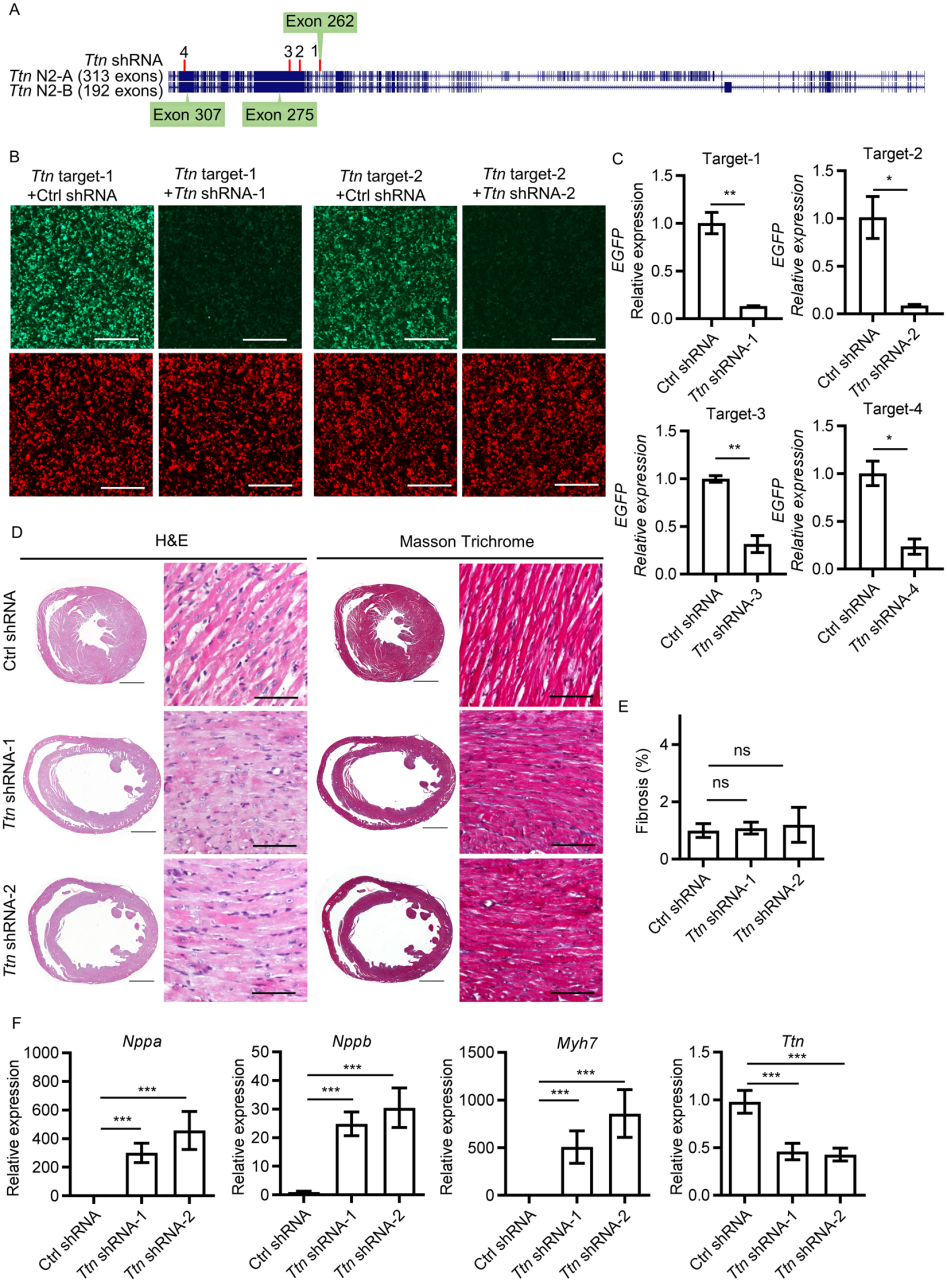
*Ttn* shRNA induces dilated cardiomyopathy in mice. (**A**) Schematic of two main isoforms of *Ttn* and the targeting sites of 4 *Ttn* shRNA candidates. Image was adapted by genome assembly of UCSC (University of California, Santa Cruz) mouse genome browser (Dec 2011)^43^. (**B**) HEK293 cells co-transfected with *Ttn* targets (Green) and corresponding shRNAs or control shRNA (Red). Magnification = 4×, scale bar = 500 µm. (**C**) Quantitative real-time PCR analysis of *EGFP* expression in HEK293 cells with two-color system. Transcription level were normalized to targets with control shRNA. n = 3. (**D**) H&E and Masson Trichrome (MT) of paraffin sections of mice 3 weeks after control shRNA, Ttn shRNA-1 and -2 transduction respectively. Virus does, 0.8E + 13 vg/kg, n ≥ 8. For completed images: magnification = 4×, scale bar = 1000 µm; for enlarged images: magnification = 20 × , scale bar = 100 µm. (**E**) Quantification of myocardial fibrosis of MT sections in (d), n ≥ 7. (**F**) Quantitative real-time PCR analysis of *Ttn*, *Nppa*, *Nppb* and *Myh7* expression, n ≥ 8.

To produce a set of *Ttn* shRNA sequences targeting *Ttn* gene expression, we designed 4 unique shRNA constructs, designated *Ttn* shRNA-1, -2, -3 or -4 (Fig. 1A). To assess their knockdown efficacy, we developed an EGFP/Cherry two-color system. Each shRNA (co-expressed with Cherry) was co-transfected with a plasmid carrying its respective target region (~500 bp) of *Ttn* gene (co-expressed with *EGFP*) into 293 T human embryonic kidney cells. Two shRNA constructs, *Ttn* shRNA-1 and -2, significantly reduced *EGFP* signal (Fig. 1B). To quantify shRNA knockdown efficacy, *EGFP* expression was measured by qPCR (Table 1). *Ttn* shRNA-1 and -2 significantly reduced *Ttn* target expression by ~ 90% (Fig. 1C). Both shRNA sequences were sub-cloned into AAV vectors (AAV-shRNA) to generate AAV-*Ttn* shRNA-1 and -2. To assess the efficacy of *Ttn* shRNA *in vivo*, we injected variable doses of AAV viruses encoding *Ttn* shRNA-1 and -2 (0.8E + 13, 2.5E + 13, and 5.0E + 13 vg/kg) into the thoracic cavity of 1.5 week old male neonates, as described^20,21^. Three weeks after viral transduction, all mice injected with medium or high dose of *Ttn* shRNA-1or -2 viruses (2.5E + 13 vg/kg, n = 4 and 5.0E + 13 vg/kg, n = 4) died due to severe heart failure. Mice treated with low dose (0.8E + 13 vg/kg, n = 8) were evaluated by echocardiography. *Ttn* shRNA-1 or -2 transduced mice reached the endpoint of showing impaired cardiac performance, enlarged left ventricle (LV) and reduced LV wall thickness (Fig. 1D). The fractional shortening (FS) of *Ttn* shRNA transduced mice (shRNA-1, 6.22 ± 1.81%, n = 8, P = 1.02E-13; shRNA-2, 4.12 ± 1.27%, n = 8, P = 2.90E-14) was significantly reduced compared to that of mice transduced with control shRNA (28.45 ± 3.80%, n = 10) (Table 2). Correspondingly, the left ventricular diastolic dimension (LVDD) (shRNA-1, 4.25 ± 0.11 mm, P = 1.75E-07; shRNA-2, 4.41 ± 0.18 mm, P = 1.67E-09, n = 8) was significantly enlarged compared to control (3.70 ± 0.12 mm, n = 10). Left ventricular wall thickness (LVWT) of *Ttn* shRNA-transduced mice (shRNA-1, 0.44 ± 0.06 mm, P = 1.60E-05, n = 8; shRNA-2, 0.41 ± 0.04 mm, P = 1.03E-06, n = 8) was significantly reduced compared to control (0.58 ± 0.05 mm, n = 10). Histological analysis did not reveal myocardial disarray in both *Ttn* shRNA-transduced mice. Moreover, cardiac fibrosis, assessed by image analysis of Masson’s trichrome (MT)-stained heart specimens and commonly observed in other DCM models including *PLN* (R9C) and *Lmna*^−/−^, was not significantly induced in *Ttn* shRNA transduced mice (shRNA-1, MT = 1.08 ± 0.21%, n = 7, P = ns; shRNA-2, MT = 1.20 ± 0.61%, n = 7, P = ns) relative to control shRNA-transduced mice (MS = 1.00 ± 0.24%, n = 9) (Fig. 1E)^6,19,22^. Instead, the expression of myocardial stress gene markers including *Nppa*, *Nppb* and *Myh7* was significantly upregulated in mice transduced with *Ttn* shRNA compared to mice transduced with control shRNA (Fig. 1F). *Ttn* shRNA *in vivo* resulted in a significant ~50% reduction of *Ttn* transcripts in *Ttn* shRNA-1 or -2 transduced mice compared to control shRNA-transduced mice. We selected *Ttn* shRNA-2 (designated as *Ttn* shRNA) for subsequent assessment. Cardiomyocytes with anti-Troponin I staining was *EGFP* positive after AAV-*EGFP* transduction, indicating the virus was distributed to most cardiomyocytes (Supplementary Fig. S1A). *Ttn* shRNA significantly reduced *Ttn* protein level (n = 5, P = 4.36E-06) (Supplementary Fig. S1B,C). These results suggest *Ttn* expression level is critical for cardiac structure and contractile function of heart and reduction of *Ttn* expression causes DCM in mice. Blast of *Ttn* shRNA targeting sequence identified potential off-target genes with at least 4 mismatch such as *Wdr9*5, *Sptbn1* and *Tnrc6b*. We did not detect a significant reduction in these gene expression after *Ttn* shRNA by qPCR (Supplementary Fig. S1D). To extend the cardiac assessment window, we further titrated virus dose of *Ttn* shRNA to 0.2E + 13 vg/kg. Thus, we could evaluate cardiac performance by additional timepoints (Table 3).

**Table 1:** The summary of qPCR primers. Primers that were designed for quantitative real-time PCR analysis were listed.

Primer	sequence	Primer	sequence
Nppa forward	tttcaagaacctgctagaccacctg	Atp2a2 forward	tgtcatcaagcacactgatcccgtc
Nppa reverse	gcttttcaagagggcagatctatcg	Atp2a2 reverse	gctgaaggggtgttctctcctgttc
Nppb forward	agtcctagccagtctccagagcaat	Esrra forward	actgcagagtgtgtggatggaagtg
Nppb reverse	cgaaggactctttttgggtgttctt	Esrra reverse	tgcacagagtcagaattggcaaggg
Ttn forward	tgcctatgtatctgggaagccacct	Cav1 forward	acacagtttcgacggcatctggaag
Ttn reverse	ggcaagaagagagtagacgccttgg	Cav1 reverse	caggaagctcttgatgcacggtaca
Myh7 forward	agcattctcctgctgtttcctt	Sod2 forward	gccacacattaacgcgcagatcatg
Myh7 reverse	tgagccttggattctcaaacg	Sod2 reverse	ccagcaactctcctttgggttctcc
Yy1 forward	cggggaataagaagtgggagcagaa	Pdgfb forward	tcttccttcctctctgctgctacct
Yy1 reverse	caggagggagtttcttgcctgtcat	Pdgfb reverse	ccccatcttcatctacggagtctct
Ccnd1 forward	atgagaacaagcagaccatccgcaa	Tmsb4x forward	atgtctgacaaacccgatatggctg
Ccnd1 reverse	cggtagcaggagaggaagttgttgg	Tmsb4x reverse	ttacgattcgccagcttgcttctct
Ccnd2 forward	tggatgctagaggtctgtgaggaac	Gja1 forward	atgggattgaagaacacggcaaggt
Ccnd2 reverse	cttggaagctaggaacatgcacact	Gja1 reverse	ccaaggacaccaccagcatgaagat
Tnrc6b forward	ataacaacagtgcctcgaaccctgg	Bcl2 forward	tctttgagttcggtggggtcatgtg
Tnrc6b reverse	actcgtgctcctccagtttcctagt	Bcl2 reverse	aaatcaaacagaggtcgcatgctgg
Wdr95 forward	ggctggatcgaatcatcagggtctg	Sirt3 forward	ctttggaggtggaggaagcagtgag
Wdr95 reverse	tgtgagttctcccttgatgccactg	Sirt3 reverse	agggtacgggatgtcatactgctga
Sptbn1 forward	tgagcatccagaactaccacctcga	Gapdh forward	aacatcatccctgcatccactggtg
Sptbn1 reverse	tctttctgcaggtcactcagcttcg	Gapdh reverse	atgcctgcttcaccaccttcttgat
Ankrd23 forward	gacacctggacattctcaaacggct	GAPDH forward	gatgacatcaagaaggtggtgaagc
Ankrd23 reverse	agtgctgtgtccccttccttatcct	GAPDH reverse	tgctgtagccaaattcgttgtcata
Fhl1 forward	actgtgtgacttgccatgagaccaa	TTN forward	gagcaagccttcagagccttcagaa
Fhl1 reverse	ccagtgatggggttcttgcatccag	TTN reverse	attccaaactcaccacgcccaagat
Abra forward	cctggttatcaatttggcccgaggt	EGFP forward	cgaaggctacgtccaggagc
Abra reverse	ttctgagtgttgtccctctccgtct	EGFP reverse	cgatgttgtggcggatcttg

**Table 2:** Effect of high dose of *Ttn* shRNA on cardiac morphology and function in mice.

Dose	Sex	Virus	Age	N	LVDD	P	LVWT	P	EF%	P	FS%	P
0.8E + 13	M	Ctrl shRNA	4.5 weeks	10	3.70 ± 0.12		0.58 ± 0.05		55.50 ± 5.51		28.45 ± 3.80	
		*Ttn* shRNA-1		8	4.25 ± 0.11	***	0.44 ± 0.06	***	14.06 ± 3.94	***	6.22 ± 1.81	***
		*Ttn* shRNA-2		8	4.41 ± 0.18	***	0.41 ± 0.04	***	9.43 ± 2.82	***	4.12 ± 1.27	***

Effect of *Ttn* shRNA on male mice at a dose of 0.8E + 13 vg/kg. P value, representing comparisons to control shRNA-transduced mice at respective age, was obtained by ANOVA and Tukey’s multiple comparisons test. LVDD, left ventricular diastolic dimension; LVWT, LV wall thickness; EF, ejection fraction; FS, fractional shortening.

**Table 3:** Effect of low dose of *Ttn* shRNA on cardiac morphology and function in mice.

Dose	Sex	Vrius	Age	N	LVDD	P	LVWT	P	EF%	P	FS%	P
0.2E + 13	M	Ctrl shRNA	4.5 weeks	7	3.70 ± 0.15		0.61 ± 0.03		59.19 ± 4.00		30.83 ± 2.68	
		*Ttn* shRNA		8	4.39 ± 0.23	***	0.46 ± 0.03	***	21.55 ± 7.92	***	9.84 ± 3.78	***
		Ctrl shRNA	5.5 weeks	7	3.88 ± 0.21		0.61 ± 0.02		54.33 ± 4.57		27.73 ± 2.97	
		*Ttn* shRNA		5	4.48 ± 0.22	**	0.48 ± 0.08	*	17.77 ± 5.12	***	7.99 ± 2.41	***
		Ctrl shRNA	6.5 weeks	7	3.80 ± 0.20		0.64 ± 0.04		56.73 ± 1.99		29.19 ± 1.22	
		*Ttn* shRNA		4	4.70 ± 0.11	***	0.48 ± 0.02	***	11.65 ± 0.69	***	5.15 ± 0.32	***

Effect of *Ttn* shRNA on male mice at a dose of 0.2E + 13 vg/kg assessed for three timepoints. P value, representing comparisons to control shRNA-transduced mice at respective age, was obtained by unpaired student T-test. LVDD, left ventricular diastolic dimension; LVWT, LV wall thickness; EF, ejection fraction; FS, fractional shortening.

### Upregulation of Yin Yang 1 (*Yy1*) suppresses DCM induced by *Ttn* shRNA

**Figure 2:**
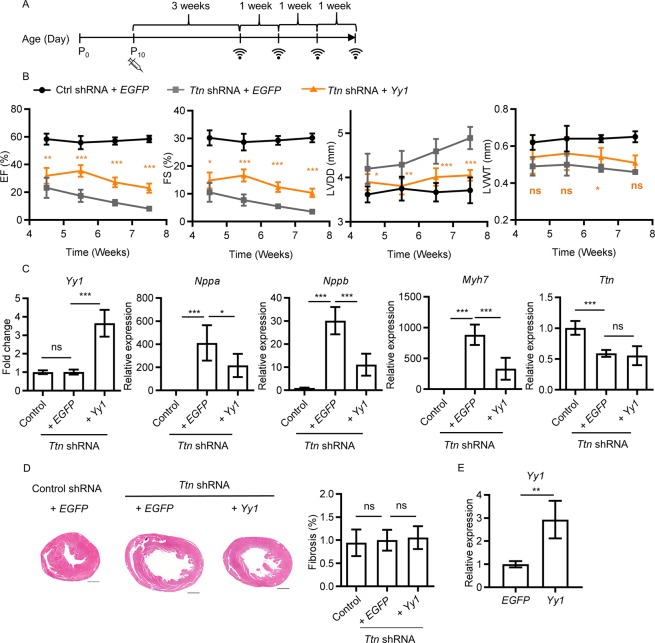
Upregulation of *Yy1* suppresses dilated cardiomyopathy induced by *Ttn* shRNA. (**A**) Experimental timeline showing timepoints of virus injection and echocardiogram. (**B**) Ejection fraction (EF), Fractional shortening (FS), left ventricular diastolic dimension (LVDD) and left ventricular wall thickness (LVWT) of mice from control group (control shRNA + *EGPF*), *Ttn* shRNA group (*Ttn* shRNA + *EGFP*) and *Yy1* treated group (*Ttn* shRNA + *Yy1*). Virus dose, 0.2E + 13 vg/kg. Cardiac performance was assessed by echocardiogram once a week from 4.5 to 7.5 weeks old. Data were presented as mean and SD, with associated P value, calculated by ANOVA and Tukey’s multiple comparisons test, representing comparison to *Ttn* shRNA group at each time point, n ≥ 8. (**C**) Quantitative real-time PCR analysis of *Yy1*, *Nppa*, *Nppb*, *Myh7* and *Ttn* expression in mice from control, *Ttn* shRNA and *Yy1* treated groups at 5.5 weeks old, n ≥ 4. (**D**) MT of Paraffin sections (left) of mice at 7.5 weeks old, magnification = 4 × , scale bar = 1000 µm, and quantification of myocardial fibrosis (right), n ≥ 4. (**E**) Quantitative real-time PCR analysis of *Yy1* expression in mice injected with control (*EGFP*) and *Yy1* respectively at 21.5 weeks old, virus dose, 0.2E + 13 vg/kg, n = 5.

To dissect the molecular mechanisms of DCM induced by *Ttn* shRNA, we profiled control (n = 3) and *Ttn* shRNA (n = 3) treated hearts (dose = 0.2E + 13 vg/kg) by RNAseq, and the significantly changed were analyzed by Gene Ontology (Supplementary Fig. S2A). We selected 8 candidates involved in downregulated categories upon *Ttn* shRNA, including *Atp2a2*, *Esrra*, *Sod2*, *Sirt3*, *Pdgfb*, *Gja1*, *Cav1* and *Tmsb4x* (Supplementary Table S1, Supplementary Fig. S2B)^23–28^. Anti-apoptosis gene *Bcl2* was included because cardiac apoptosis exhibits in DCM models^29,30^. In addition, we added *Yy1* which significantly enhanced *TTN* gene expression when co-transduced together with 5 factors (*GATA4*, *MEF2C*, *TBX5*, *HAND2* and *MYOCD*, designated as 5 F) in human dermal fibroblasts (Supplementary Fig. S3). All 10 candidates were constructed into an AAV cis-vector (AAV-cTnT) transcriptionally controlled by cardiac Troponin T promoter. After each candidate (0.2E + 13 vg/kg) was co-introduced with *Ttn* shRNA (0.2E + 13 vg/kg), cardiac performance was assessed by echocardiography. When treated with most selected candidates, *Ttn* shRNA transduced mice developed a similar or even more severe DCM compared to those treated with control cDNA (0.2E + 13 vg/kg, expressing *EGFP* under cardiac troponin T promoter, designated as *EGFP* control), indicating that re-introducing these pathway-associated candidates could not suppress *Ttn* shRNA-induced DCM (Supplementary Table S2). Conversely, *Yy1* (FS = 16.39 ± 1.44%, n = 5, P = 1.26E-03) significantly improved cardiac performance of *Ttn* transduced mice compared to those treated with *EGFP* control (FS = 9.91 ± 4.32%, n = 20).We further assessed the suppressive effect of *Yy1* on DCM induced by *Ttn* shRNA for three additional timepoints (Fig. 2A,B, Supplementary Table S3). Three weeks after virus transduction, cardiac chamber dilation (LVDD) of *Ttn* shRNA transduced mice treated by *Yy1* (3.90 ± 0.18 mm, n = 8, P = 4.20E-02) was significantly suppressed compared to that treated by *EGFP* control (4.20 ± 0.34 mm, n = 12). Remarkably, *Yy1* treated mice continued to maintain significantly rescue of the cardiac chamber dilation compared to *Ttn* shRNA transduced mice treated by *EGFP* control. Cardiac function (FS) of *Ttn* shRNA mice treated with *Yy1* was significantly improved compared to *Ttn* shRNA mice treated by *EGFP* control, suggesting that *Yy1* suppresses DCM induced by *Ttn* shRNA. At dose of 0.2E + 13 vg/kg, candidate *Yy1* was induced ~ 4.5 fold by qPCR analysis. The expression of heart failure markers including *Nppa*, *Nppb* and *Myh7* in mice treated with *Yy1* was significantly reduced compared to that in DCM mice treated with *EGFP* control (Fig. 2C). *Ttn* shRNA significantly reduced *Ttn* expression and protein level which were not induced by upregulation of *Yy1*, suggest *Yy1* does not regulate *Ttn* in mouse hearts (Fig. 2C, Supplementary Fig. S4). Importantly, we did not observe increased fibrosis by *Yy1* (Fig. 2D). Upregulation of *Yy1* in wildtype mice did not alter cardiac performance at least 5 months post virus transduction, when ~3.0 fold increase of *Yy1* expression was detected by qPCR analysis (Supplementary Table S4, Fig. 2E).

### Upregulation of Yy1 modulates cardiac structural contractile and cell growth related gene expression

**Figure 3:**
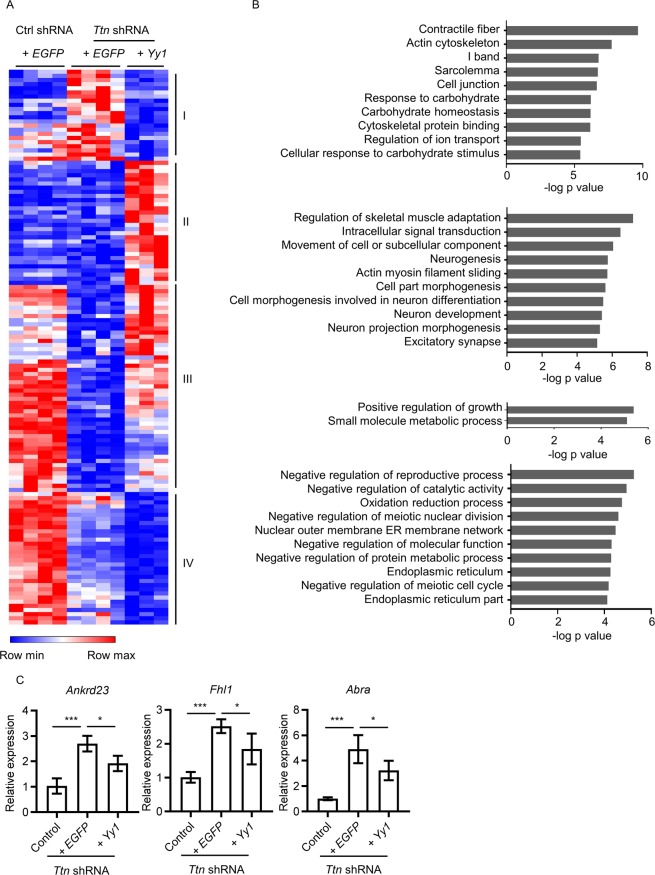
Upregulation of *Yy1* modulates cardiac structural contractile, cell proliferation and survival related gene expression. (**A**) Heat map representing color-coded expression level of 134 genes that significantly changed in *Yy1* treated group (*Ttn* shRNA + *Yy1*) in comparison to *Ttn* shRNA group (*Ttn* shRNA + *EGFP*). Virus does, 0.2E + 13 vg/kg. Mice were harvest four weeks after transduction, n ≥ 3. (**B**) Top10 gene sets of GO of each category by GSEA, arranged by −log P value. (**C**) Quantitative real-time PCR analysis of *Ankrd23*, *Fhl1*, *Abra* expression in control, *Ttn* shRNA and *Yy1* treated group, n ≥ 3.

To dissect the molecular mechanisms of *Yy1* suppressing *Ttn* shRNA-induced DCM, we profiled control shRNA + *EGFP* (designated as control shRNA group, male, n = 4, dose = 0.2E + 13 vg/kg), *Ttn* shRNA + *EGFP* (designated as *Ttn* shRNA group, male, n = 4, dose = 0.2E + 13 vg/kg) and *Ttn* shRNA + *Yy1* animals (designated as *Yy1* treated group, male, n = 3, dose = 0.2E + 13 vg/kg) four weeks after virus transduction by RNAseq. To uncover signaling pathways regulated by *Yy1*, we found 134 genes significantly changed in *Yy1* treated group compared to *Ttn* shRNA group (Fig. 3A). Among them, 80 genes were elevated, and 54 genes were reduced by *Yy1*. Hierarchical clustering of control shRNA, *Ttn* shRNA and *Yy1* groups built significantly dysregulated genes into 4 regulated patterns. We analyzed gene list by Gene Set Enrichment Analysis (GSEA, Broad Institute). Gene ontology (GO) analysis identified top significantly enriched gene sets in category III including “positive regulation of growth” and “small molecule metabolic process” (Fig. 3B). Contractile fiber GO in category I included genes associated with sarcomere, such as *Ankrd23*, *Fhl1* and *Abra*. The upregulation of four-and-a-half LIM domain protein 1 (*Fhl1*) and Ankyrin repeat domain-containing protein 23 (*Ankrd23*) by *Ttn* shRNA was suppressed by *Yy1* (Fig. 3C). Importantly, Fhl1 and Ankrd23 interacted with *Ttn* protein, suggesting *Yy1* modulates *Ttn* associated partners^31,32^. These results indicated *Yy1* modulates *Ttn* partners and cardiac cell growth related gene expression which contributing to repair cardiac function.

### Upregulation of *Yy1* promotes cardiomyocyte cell cycle in dilated cardiomyopathy induced by *Ttn* shRNA

**Figure 4:**
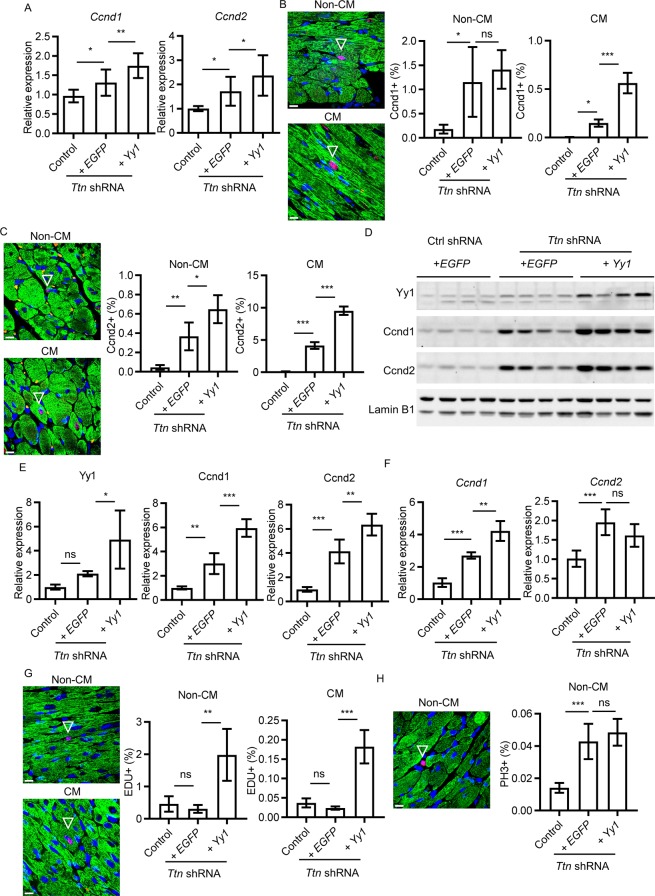
Upregulation of *Yy1* promotes cardiac cell cycle in dilated cardiomyopathy induced by *Ttn* shRNA. (**A**) Quantitative real-time PCR analysis of *Ccnd1* and *Ccnd2* expression in mouse heart tissue from control, *Ttn* shRNA and *Yy1* treated groups. Virus does, 0.2E + 13 vg/kg. Mice were harvest four weeks after transduction. n ≥ 10. (**B**) Paraffin sections (left) stained with DAPI (blue), cTnI (green) and Ccnd1 (red), representing Non-CM and CM with positive Ccnd1 signal (arrowed); quantification of Ccnd1 + Non-CM and CM (right) from control, *Ttn* shRNA and *Yy1* treated groups, n ≥ 4. (**C**) Paraffin section (left) and quantification (right) of Ccnd2 + in Non-CM and CM respectively, n ≥ 4. (**D**) Western blot and quantitative analysis (**E**) of Yy1, Ccnd1 and Ccnd2 protein levels in mouse heart tissue, n = 4. Data were normalized by Lamin B1 antibody. Full-length blots were presented in Supplementary Fig. S6. (**F**) Quantitative real-time PCR analysis of *Ccnd1* and *Ccnd2* expression in isolated CM from control, *Ttn* shRNA and *Yy1* treated groups four weeks after virus transduction, n ≥ 3. (**G**) Paraffin section (left) and quantification (right) of EdU + in Non-CM and CM respectively, n = 4. (**H**) Paraffin section (left) and quantification (right) of pH3 + in non-CM, n ≥ 4. In (**B**), (**C**), (**G**) and (**H**), magnification = 100×, scale bar = 10 µm. Data were shown as mean ± SD and were normalized to total nucleus number labelled by DAPI.

Because regulation of cell growth pathway was significantly enriched comparing *Yy1* treated group to *Ttn* shRNA group by RNAseq analysis, we assessed cell cycle reentry genes including *Ccnd1* and *Ccnd2* by qPCR. *Ccnd1* expression was significantly induced by ~1.36 fold (n = 12, P = 2.50E-02) in *Ttn* shRNA group compared to control shRNA group (n = 10). *Ccnd2*, another cyclin family member, was significantly induced by ~1.71 fold (n = 12, P = 1.92E-02) in *Ttn* shRNA group compared to control shRNA group (n = 10). *Yy1* significantly enhanced additional *Ccnd1* and *Ccnd2* expression by ~ 1.33 fold (n = 11, P = 3.07E-03) and ~1.38 fold (n = 11, P = 3.58E-02) respectively compared to *Ttn* shRNA group (n = 12) (Fig. 4A). To locate *Ccnd1* and *Ccnd2* expression in heart tissues, we stained heart sections with Ccnd1 or Ccnd2 antibody together with an antibody recognizing cardiac Troponin I (cTn I) for cardiomyocytes (CM). *Ccnd1* protein was predominantly detected in the nuclei of non-cardiomyocytes (non-CM) (Supplementary Fig. S5A). We observed a significant increase of non-CM positive for Ccnd1 (Ccnd1+) in *Ttn* shRNA group (1.16 ± 0.72%, n = 4, P = 2.38E-02) compared to control group (0.18 ± 0.09%, n = 5) (Fig. 4B). Importantly, a significant ~ 39.86 fold of Ccnd1 + CM (0.15 ± 0.04%, n = 4, P = 1.36E-02) in *Ttn* shRNA group was increased compared to those in control shRNA group (0.0038 ± 0.0026%, n = 5), suggesting that *Ttn* shRNA activates CM cell cycle reentry. In addition, Ccnd1 + CM was further increased by ~3.76 fold (0.56 ± 0.11%, n = 4, P = 7.02E-06) in *Yy1* treated group compared to *Ttn* shRNA group. *Ccnd2* protein was predominantly expressed and elevated in the nuclei of CM (Supplementary Fig. S5B). In comparison to control group (0.11 ± 0.03%, n = 5), we detected significantly ~ 37.20 fold more CM positive for Ccnd2 (Ccnd2+) in *Ttn* shRNA group (4.14 ± 0.52%, n = 4, P = 3.21E-07) (Fig. 4C). *Yy1* significantly increased additional Ccnd2 + CM by~2.30 fold (9.53 ± 0.64%, n = 4, P = 3.48E-08) compared to *Ttn* shRNA group. We also observed a significant increase of Ccnd2 + non-CM in *Ttn* shRNA group (0.37 ± 0.14%, n = 4, P = 4.56E-03) compared to controls (0.05 ± 0.02%, n = 5), and *Yy1* further significantly increased Ccnd2 + non-CM (0.65 ± 0.14%, n = 4, P = 1.36E-02) compared to *Ttn* shRNA group. We quantified protein levels of *Ccnd1* and *Ccnd2* in heart tissues by western blot analysis (Fig. 4D, Supplementary Fig. S6). Upon *Ttn* shRNA, we detected a significant ~ 3.01 fold (n = 4, P = 4.78E-03) and ~ 4.14 fold (n = 4, P = 7.46E-04) increase of Ccnd1 and Ccnd2 proteins compare to control shRNA (n = 4). In comparison to *Ttn* shRNA group (n = 4), additional ~ 1.98 fold (n = 4, P = 3.52E-04) and ~ 1.53 fold (n = 4, P = 7.45E-03) of Ccnd1 and Ccnd2 was increased in *Yy1* group (Fig. 4E). We further examined how cyclin D was regulated in CM. *Ccnd1* and *Ccnd2* was assayed in isolated CM by qPCR as previous described^33^. *Ccnd1* and *Ccnd2* expression was increased by ~ 2.63 fold (2.71 ± 0.2%, n = 3, P = 7.35E-04) and ~ 1.93 fold (1.96 ± 0.33%, n = 3, P = 9.95E-04) in CM derived from *Ttn* shRNA group compared to control shRNA group (1.03 ± 0.27%, 1.02 ± 0.21%, n = 5), suggesting CM activated cell cycle reentry by upregulating *Ccnd1* and *Ccnd2* expression in response to *Ttn* insufficiency (Fig. 4F). *Yy1* significantly enhanced additional *Ccnd1* expression by ~ 1.56 fold (4.22 ± 0.62%, n = 3, P = 2.86E-03) compared to *Ttn* shRNA group (n = 3). *Yy1* did not further increase *Ccnd2* expression, suggesting *Ccnd2* is not transcriptionally regulated by *Yy1* in CM. To assess whether activated cardiac cell cycle advanced to G1/S phase, we assayed DNA synthesis by EdU incorporation. Most EdU was detected in non-CM because they do not exit from cell cycle (Supplementary Fig. S5C). We observed a comparable level of CM DNA positive for EdU (EdU + ) between control group (0.04±0.01%, n = 4, P = ns) and *Ttn* shRNA group (0.02 ± 0.004%, n = 4), suggesting CM cell cycle activated by endogenously upregulated genes including *Ccnd1* and *Ccnd2* does not advance to S phase (Fig. 4G). Upon *Yy1* treatment, ~ 7.63 fold (0.18 ± 0.04%, P = 3.03E-05, n = 4) increase of EdU + CM was detected compared to *Ttn* shRNA group, indicating *Yy1* promoted CM cell cycle from G1 to S phase. EdU incorporation also was increased by ~ 6.41 fold in non-CM of *Yy1* group. To assess whether cardiac cell cycle further progressed to mitotic phase, we stained heart sections with M phase marker, phosphorylated histone (pH3) (Supplementary Fig S5D). We observed an elevated number of pH3 positive (pH3+) non-CM in *Ttn* shRNA group (0.04 ± 0.01%, n = 4, P = 6.78E-04) compared to control shRNA group (0.01 ± 0.003%, n = 5). Although *Yy1* group showed an increased EdU incorporation in non-CM, upregulation of *Yy1* did not further increase pH3 + signals (0.05 ± 0.01%, n = 4, P = ns) in non-CM of *Yy1* group compared to *Ttn* shRNA group, suggesting upregulation of *Yy1* does not increase non-CM mitosis. We did not observed pH3 + CM in the hearts of all three groups, indicating activated CM reaching to M phase are limited (Fig. 4H).

### Upregulation of *Ccnd1* and *Ccnd2* suppresses DCM induced by *Ttn* shRNA

*Ccnd1* and *Ccnd2* was significantly enhanced by *Yy1* in CM of mouse hearts. To assess whether enhancing of Cyclin D suppresses DCM caused by *Ttn* insufficiency, we co-introduced *Ccnd1* or *Ccnd2* (0.2E + 13 vg/kg) with *Ttn* shRNA (0.2E + 13 vg/kg). Cardiac performance was assessed by echocardiography. Cardiac function (FS) of mice treated with *Ccnd1* or *Ccnd2* was significantly improved compared to *Ttn* shRNA mice treated by *EGFP*. *Ccnd1* or *Ccnd2* overexpression significantly suppressed DCM induced by *Ttn* shRNA, suggesting cyclin D serves as a crucial downstream of *Yy1* to enhance cardiac performance (Supplementary Table S5).

## Discussion

*Ttn* truncating variants are enriched in DCM patients suggesting a causal effect of *TTN* variants on DCM^3^. *TTN* Haploinsufficiency caused by TTNtv is emerging as the disease mechanism. To assess whether *Ttn* insufficiency causes DCM, we used shRNA to modulate *Ttn* expression. By reducing ~ 50% of *Ttn* expression, we generated a mouse model demonstrating a severe DCM phenotype, including ventricular wall thinning, dilated ventricular chambers and impaired cardiac function. To develop potential therapy for suppressing DCM caused by *Ttn* insufficiency, we screened 10 genes involved in different pathways. As a transcriptional regulator of *TTN* during direct cardiac reprogramming, we included *Yy1* for the rescue experiments. *Ttn* expression was not induced by *Yy1* in DCM hearts caused by *Ttn* shRNA, suggesting that *Yy1* does not inhibit DCM through *Ttn* gene regulation in mouse hearts. Recently, *Yy1* was shown to promote both *Nkx2*.*5* expression, cardiac progenitor cell commitment and maintenance during early embryo development^34,35^. Cardiomyocyte-specific ablation of *Yy1* mediated by *Myh6*-cre resulted in perinatal death of mutant mice, suggesting *Yy1* plays an important role in early and late cardiac lineage development^36^. Importantly, *YY1* is shown to be upregulated in human idiopathic dilated cardiomyopathy (IDC) and heart failure^37,38^.

We detected over 35 fold increase of Ccnd1 and Ccnd2 in cardiomyocyte nuclei upon *Ttn* shRNA, indicating cardiomyocytes response to *Ttn* insufficiency by activating cell cycle reentry signals. We did not observed this reaction in another DCM model caused by *Lmna* insufficiency. Activated cardiomyocyte cell cycle induced by *Ttn* insufficiency does not advance to S phase. We found that upregulation of *Yy1* promoted cardiac cell cycle reentry by further enhancing *Ccnd1* and *Ccnd2*. *Yy1* promoted cardiomyocyte cell cycle to S phase by a significant increase of EdU incorporation. However, we did not detect mitotic phase marker pH3 in cardiomyocytes, suggesting activated cardiomyocytes undergoing mitotic phase are limited. Importantly, Upregulation of *Ccnd1* and *Ccnd2* suppressed DCM caused by *Ttn* insufficiency. Taken together, *Yy1* promotes cardiac cell cycle to facilitate to suppress *Ttn* shRNA-induced DCM.

Our recent study showed insufficiency of *Mybpc3*, another causal gene for sarcomeric cardiomyopathy, induces an extra round of cardiomyocyte cell cycle during neonatal stage^19^. It is now of great interest to know whether this reactivation of cardiac cell cycle is a common mechanism for sarcomere deficiency and enhancing of this process by *Yy1* or other regulators is able to suppress cardiovascular disease related to sarcomere. Reactivation of cardiomyocyte cell cycle does not always lead to cell number increase. Cytokinesis is still rarely detected in adult hearts upon regenerative enhancement. *Yy1* might promote hypertrophic growth coupled with cell cycle reentry as *Ccnd2* is a mediator for *Myc* overexpression or exercise-induced hypertrophic growth in cardiomyocytes^39^. Recently, *Yy1* is suggested to serve as a structural regulator between enhancer and promoter interactions and facilitates gene expression^40^. Apart from modulating cell growth, upregulation of *Yy1* might indeed reinstate or promote enhancer - promoter interaction to restore gene regulatory network dysregulated by *Ttn* insufficiency.

Our selected candidates included many therapeutic targets for heart failure including *Atp2a2*, *Bcl2*, *Sod2* and *Sirt3*^23,24,28,41^. Most of them failed to protect DCM induced by *Ttn* shRNA, suggesting root causes and disease mechanisms should be taken into account for DCM prevention and treatment strategies. It is of great interest to know whether *Yy1* is able to suppress DCM caused by other genes including *LMNA*, *MYH7* and *PLN*. To translate our research, one concern is whether upregulation of *Yy1* could cause any cardiac defects. Previous study showed overexpression of *Yy1* induced a relative marginal hypertrophy cardiomyopathy only in male mice. Here, we specified the *Yy1* expression by cardiac specific promoter, virus dose and postnatal transduction. In contrast to a previous study, no detectable impairment of cardiac structure and performance was observed after ~ 5 months of virus transduction^37^. Taken together, our findings provide a strong supporting evidence for translational research.

## Materials and Methods

### Animal protocols

All mice were maintained and studied using protocols approved by the Institutional Animal Care and Use Committee (IACUC) of National University of Singapore. Animal work was undertaken in accordance with Singapore National Advisory Committee for Laboratory Animal Research guidelines. Relevant national and institutional guidelines and regulations must be consulted before commencement of any animal work. All studies were conducted in male C57BL/6JINV (Jax) mice. For virus injection, 50 µl viruses were injected into thoracic cavity of 10 days old pups via insulin syringe, avoiding the heart and lungs. For EdU injection, EdU (Sigma, 900584) was dissolved in saline and 5 mg/kg EdU was delivered to mice by intraperitoneal injection for two weeks after AAV transduction. For heart harvesting, mouse was anesthetized by 2% isoflurane and the heart was exposed by opening chest. After that, 15% KCl was injected into inferior vena cava to achieve asystole at diastole, then the heart was rapidly isolated and flushed with D-PBS through LV to wash out blood. Half of the apex was isolated and immersed in RNALater (Qiagen, 76104) at room temperature for RNA extraction, while the other half was snap frozen in liquid nitrogen for protein extraction. The rest part of heart was fixed in 4% paraformaldehyde for 24 hours and subsequently embedded by paraffin.

### Echocardiogram (Echo) and surface electrocardiogram (ECG)

Cardiac dimension and function of mice three and/or four weeks after virus transduction were achieved by echocardiography (VisualSonics, Vevo 2100, 40 Mhz-550S probe). All mice were shaved to expose chest area one day before experiment. During echo, 1.5% isoflurane with oxygen were applied to each mouse, and cine of 300 frames of both B mode and M mode (left parasternal long and short axes) were recorded when heart rate was around 450–500 bpm. Measurements were processed by Vevo®LAB (VisualSonics Inc.). LV tracings were averaged from at least 3 consecutive heart beats of M-mode. LVDD (LV diastolic dimensions), LVWT (LV posterior wall thickness), EF (ejection fraction) and FS (fractional shortening) were obtained from short axis images.

### Cell culture and transfection

HEK293T cells were cultured at 37 °C with 5% CO2 and maintained in DMEM (Hyclone) supplemented with 10% FBS, 1 mM sodium pyruvate and 10 µg/ml gentamicin. Transfection of shRNA constructs and other plasmids was performed using PEI (Polysciences. Inc, 24765-2) according to manufacturer’s instructions.

### Cardiomyocyte isolation

We adapted protocol from Ackers-Johnson *et al*. In brief, the heart was perfused with warm EDTA, perfusion buffer and collagenase buffer through LV when aorta was clamped, then heart tissue became softened and swollen. After that, heart tissue was teared into pieces and dissociated by pipetting. Cell suspension was then passed through 100 µm strainer and processed to three rounds of gravity settling. Cardiomyocytes, in the pellet, were harvested for RNA extraction.

### Recombinant adeno-associated viruses 9 (rAAV9) production, purification and titration

Protocol was adapted from Grieger *et al*. In brief, rAAVs were produced on HEK293T cells by transient triple plasmid transfection, including rAAV viral vector with gene to be delivered, helper plasmids pAdΔF6 and plasmid pAAV2/9 (Penn Vector Core). Three days after transfection, viruses were collected from cell pellets and were purified by Optiprep density gradient medium (Sigma, D-1556). After concentrating by centrifugal filter (Milipore, UFC910096), viruses were aliquoted and stored at −80 °C. For virus titration, forward primer: gataaaagcagtctgggctttcaca and reverse primer: gagcccatataagcccaagctattg were designed to target at rAAV genome-containing particle, cTnT promotor region, and to determinate the physical titers by qPCR.

### Lentiviral production and transduction

Lentiviral plasmids teto-HAND2, FUW-TetO-GATA4, teto-MEF2C, teto-TBX5, teto-MYOCD and teto-YY1were obtained from Addgene or by cloning. Lentiviruses were produced on HEK293T cells. Two days after transfection, viruses were harvested from supernatant and concentrated by centrifugal filter (Milipore, UFC910096). Lenti-X^TM^ qRT-PCR Titration Kit was applied for titration. As for virus infection, human primary fibroblasts (ATCC PCS-201-010) were subjected to two rounds of overnight transduction at 50% confluency.

### shRNA vector construction

*Ttn* shRNA candidates were designed to target 21 base-pair gene-specific regions (Invitrogen). The sequences of mouse *Ttn* shRNAs are as follows: shRNA-1: AGTACTTTCAGCTTAATGGTG (Exon 262); shRNA-2: TAAAGAAGCCGATTTCTTGGT (Exon 275); shRNA-3: ATTACTGGCACACTCAGTTGT (Exon 275); shRNA-4: ATAAGTTGGAGACAAGGAGCG (Exon 307). The sequence against LacZ, AAATCGCTGATTTGTGTAGTC, was used as control shRNA. The miR-155 backbone based shRNA cassette was inserted at 3 prime of AAV-*cTnT*-*EGFP* vector.

### RNAseq library preparation and next generation sequencing

Total RNA from left ventricular tissue of male mice (n = 3 per group) was achieved to establish RNAseq library. RNA samples were pre-treated with Truseq Stranded Total RNA Library Prep kit (Illumina, RS-122–2201) to remove abundant cytoplasmic rRNA. The remaining intact RNA was fragmented using a chemical mix, followed by first- and second-strand cDNA synthesis using random hexamer primers. End-repaired fragments were ligated with a unique illumina adapter. All individually indexed samples were subsequently pooled together and multiplexed for sequencing. Libraries were sequenced using the Illumina Hiseq. 2000 sequencing system and paired-end 101 bp reads were generated for analysis. RNAseq data was deposited to NCBI. For selecting potential therapeutic genetic candidates, 3101 genes (log fold change <−0.59 or >0.7, FDR <0.05) were identified from DCM group (*Ttn* shRNA) compared to control group (Ctrl shRNA). For dissecting molecular mechanism of Yy1, 134 genes (P < 0.005 and FDR <0.2) were selected from *Yy1* treated group (*Ttn* shRNA + *Yy1*) compared to *Ttn* shRNA group (*Ttn* shRNA + *EGFP*). Differentially expressed genes were uploaded to Morpheus for Hierarchical clustering and color-coded heat-map. Gene ontology (GO) of each category were analyzed by Gene Set Enrichment Analysis (GSEA, Broad Institute).

### Quantitative real-time PCR (qPCR)

Transcription level were quantified by qPCR. cDNA was synthesized using Maxima First Strand kit (ThermoFisher, K1641) and qPCR was carried out by KAPA SYBR Fast qPCR Master Mix kit (KAPA Biosystems, KR0389). All qPCR primers are listed as follows:

### Histological and immunostaining analysis

Heart samples were fixed in 4% paraformaldehyde for 24 hours, then embedded in paraffin and sectioned at 5 µm intervals. Paraffin samples were further treated with xylene (to remove paraffin), re-hydrated, and permeabilized in 0.1%(v/v) Triton-X100 in PBS. Hematoxylin and eosin (HE) was applied to observe myocyte architecture and Masson trichrome (MT) to identify cardiac fibrosis. Fibrosis was quantified by Image J. The percentage of fibrosis was calculated as the blue-stained areas divided by total ventricular area. As for immunostaining, boiled citric acid was used for Ccnd1, Ccnd2, EdU and pH3. Primary antibodies: Ccnd1 (Abcam, ab16663), Ccnd2 (Cell Signaling Technology, 3741), pH3 (Cell Signaling Technology, 9718) and cTnI (Abcam, ab8295). Other labeling dyes: DAPI for nucleus (ThermoFisher, D1306) and EdU Imaging kit (ThermoFisher, C10229). All the positive signals from three completed cross sections were counted for each heart sample, and data were normalized to total nucleus number.

### Western blots

Frozen heart tissues were lysed in cold RIPA buffer with protease inhibitor (Sigma, 4693116001) and were homogenized with prechilled TissueLyser (Qiagen, 25/s, 2 mins, 3 cycles) by adding metal beads. After centrifuge, supernatants were collected to detect proteins with small molecular weight like Yy1, Ccnd1 and Ccnd2. 20 µg of each sample was loaded into SDS-page gel and further transfer to nitrocellulose membrane (0.2 µm, Biorad, 162-0112) for blotting. Primary antibody including: Yy1 (Thermofisher, PA5-29171), Ccnd1 (Abcam, ab16663), Ccnd2 (Cell Signaling Technology. Inc, 3741), and Lamin B1 (Abcam, ab16048). Secondary antibody including: Donkey anti-Rabbit IgG (H + L) Highly Cross-Adsorbed Secondary Antibody (Thermofisher, A16035).

### Coomassie blue staining for *Ttn* protein

As for pellet fraction, protocol was adapted from Neagoe *et al*.^42^. Briefly, pellets were resuspended in solubilization buffer (1% SDS, 1% 2-mercaptoethanol, 10% glycerol, 8 µg/ml leupeptin, 4.3 mM Tris–HCl, pH 8.8, 4.3 mM EDTA) and samples were further incubated on ice for 10 mins followed by boiling (95 °C) for 3 mins. 35 µg protein from each sample was added with 6 µM of bromophenol blue (Sigma, 114391) and then loaded onto agarose-strengthened SDS-PAGE gel (3.6% polyacrylamide, 1% agarose with a laemmli buffer system). Protein bands were visualized by SimplyBlue SafeStain (Life technologies, LC6060) and band intensity were quantified using ImageLab (Bio-Rad).

### Statistical analyses

Statistical analysis was achieved by Prism Graphpad 7.0. P value between two groups was performed by two-tailed, unpaired T-test with Weltch correction, and one-way ANOVA with Tukey’s multiple comparisons test for multiple groups. Quantitative data were shown as mean ± SD. ns, non-significant, P > 0.05, *P < 0.05, **P < 0.01, ***P < 0.001.

## Supplementary information

SUPPLEMENTARY INFO

## Data Availability

The datasets generated during and/or analysed during the current study are available in the NCBI repository, https://www.ncbi.nlm.nih.gov/Traces/study/?acc=PRJNA565836.

## References

[1] Hershberger RE, Hedges DJ, Morales A (2013). Dilated cardiomyopathy: the complexity of a diverse genetic architecture. Nat Rev Cardiol.

[2] Weintraub RG, Semsarian C, Macdonald P (2017). Dilated cardiomyopathy. Lancet.

[3] Herman DS (2012). Truncations of titin causing dilated cardiomyopathy. N Engl J Med.

[4] Fatkin D (1999). Missense mutations in the rod domain of the lamin A/C gene as causes of dilated cardiomyopathy and conduction-system disease. N Engl J Med.

[5] Olson TM (2000). Inherited and de novo mutations in the cardiac actin gene cause hypertrophic cardiomyopathy. J Mol Cell Cardiol.

[6] Schmitt JP (2003). Dilated cardiomyopathy and heart failure caused by a mutation in phospholamban. Science.

[7] McNally EM, Golbus JR, Puckelwartz MJ (2013). Genetic mutations and mechanisms in dilated cardiomyopathy. J Clin Invest.

[8] Ware JS (2016). Shared Genetic Predisposition in Peripartum and Dilated Cardiomyopathies. N Engl J Med.

[9] Gerull B (2002). Mutations of TTN, encoding the giant muscle filament titin, cause familial dilated cardiomyopathy. Nat Genet.

[10] Schafer S (2017). Titin-truncating variants affect heart function in disease cohorts and the general population. Nat Genet.

[11] Pasumarthi KB, Field LJ (2002). Cardiomyocyte cell cycle regulation. Circ Res.

[12] Bergmann O (2009). Evidence for cardiomyocyte renewal in humans. Science.

[13] Senyo SE (2013). Mammalian heart renewal by pre-existing cardiomyocytes. Nature.

[14] Morikawa Y, Heallen T, Leach J, Xiao Y, Martin JF (2017). Dystrophin-glycoprotein complex sequesters Yap to inhibit cardiomyocyte proliferation. Nature.

[15] Mahmoud AI (2013). Meis1 regulates postnatal cardiomyocyte cell cycle arrest. Nature.

[16] See K (2017). Single cardiomyocyte nuclear transcriptomes reveal a lincRNA-regulated de-differentiation and cell cycle stress-response *in vivo*. Nat Commun.

[17] Chen J (2013). mir-17-92 cluster is required for and sufficient to induce cardiomyocyte proliferation in postnatal and adult hearts. Circ Res.

[18] Lin Z (2014). Cardiac-specific YAP activation improves cardiac function and survival in an experimental murine MI model. Circ Res.

[19] Jiang J (2015). Cardiac myosin binding protein C regulates postnatal myocyte cytokinesis. Proc Natl Acad Sci USA.

[20] Jiang J, Wakimoto H, Seidman JG, Seidman CE (2013). Allele-specific silencing of mutant Myh6 transcripts in mice suppresses hypertrophic cardiomyopathy. Science.

[21] Wakimoto H, Seidman JG, Foo RS, Jiang J (2016). AAV9 Delivery of shRNA to the Mouse Heart. Curr Protoc Mol Biol.

[22] Wolf CM (2008). Lamin A/C haploinsufficiency causes dilated cardiomyopathy and apoptosis-triggered cardiac conduction system disease. J Mol Cell Cardiol.

[23] Nelson BR (2016). A peptide encoded by a transcript annotated as long noncoding RNA enhances SERCA activity in muscle. Science.

[24] Zangi L (2013). Modified mRNA directs the fate of heart progenitor cells and induces vascular regeneration after myocardial infarction. Nat Biotechnol.

[25] Pasumarthi KB, Nakajima H, Nakajima HO, Soonpaa MH, Field LJ (2005). Targeted expression of cyclin D2 results in cardiomyocyte DNA synthesis and infarct regression in transgenic mice. Circ Res.

[26] Van Remmen H (2001). Knockout mice heterozygous for Sod2 show alterations in cardiac mitochondrial function and apoptosis. Am J Physiol Heart Circ Physiol.

[27] Hafner AV (2010). Regulation of the mPTP by SIRT3-mediated deacetylation of CypD at lysine 166 suppresses age-related cardiac hypertrophy. Aging (Albany NY).

[28] Chatterjee S (2002). Viral gene transfer of the antiapoptotic factor Bcl-2 protects against chronic postischemic heart failure. Circulation.

[29] Nikolova V (2004). Defects in nuclear structure and function promote dilated cardiomyopathy in lamin A/C-deficient mice. J Clin Invest.

[30] Crone SA (2002). ErbB2 is essential in the prevention of dilated cardiomyopathy. Nat Med.

[31] Raskin A (2012). A novel mechanism involving four-and-a-half LIM domain protein-1 and extracellular signal-regulated kinase-2 regulates titin phosphorylation and mechanics. J Biol Chem.

[32] Miller MK (2003). The muscle ankyrin repeat proteins: CARP, ankrd2/Arpp and DARP as a family of titin filament-based stress response molecules. J Mol Biol.

[33] Ackers-Johnson M (2016). A Simplified, Langendorff-Free Method for Concomitant Isolation of Viable Cardiac Myocytes and Nonmyocytes From the Adult Mouse Heart. Circ Res.

[34] Gregoire S (2013). Essential and unexpected role of Yin Yang 1 to promote mesodermal cardiac differentiation. Circ Res.

[35] Gregoire S, Li G, Sturzu AC, Schwartz RJ, Wu SM (2017). YY1 Expression Is Sufficient for the Maintenance of Cardiac Progenitor Cell State. Stem Cells.

[36] Beketaev I (2015). Critical role of YY1 in cardiac morphogenesis. Dev Dyn.

[37] Stauffer BL (2015). Transgenic over-expression of YY1 induces pathologic cardiac hypertrophy in a sex-specific manner. Biochem Biophys Res Commun.

[38] Sucharov CC, Mariner P, Long C, Bristow M, Leinwand L (2003). Yin Yang 1 is increased in human heart failure and represses the activity of the human alpha-myosin heavy chain promoter. J Biol Chem.

[39] Zhong W (2006). Hypertrophic growth in cardiac myocytes is mediated by Myc through a Cyclin D2-dependent pathway. EMBO J.

[40] Weintraub AS (2017). YY1 Is a Structural Regulator of Enhancer-Promoter Loops. Cell.

[41] Kawase Y (2008). Reversal of cardiac dysfunction after long-term expression of SERCA2a by gene transfer in a pre-clinical model of heart failure. J Am Coll Cardiol.

[42] Neagoe C, Opitz CA, Makarenko I, Linke WA (2003). Gigantic variety: expression patterns of titin isoforms in striated muscles and consequences for myofibrillar passive stiffness. J Muscle Res Cell Motil.

[43] Kent WJ (2002). The human genome browser at UCSC. Genome Res..

