# Supplementary material for: Upregulation of Yy1 Suppresses Dilated Cardiomyopathy caused by Ttn insufficiency
Source: Sci Rep. 2019 Nov 8;9:16330. doi: 10.1038/s41598-019-52796-0 (PMC6841687; doi:10.1038/s41598-019-52796-0)
Supplement: Supplementary file 1 — SUPPLEMENTARY INFO [file 41598_2019_52796_MOESM1_ESM.docx]

**Title**

Upregulation of *Yy1* Suppresses Dilated Cardiomyopathy caused by *Ttn* insufficiency

**Authors**

Dan Liao^1,2^ , Weiming Chen^1,2^, Chia Yee Tan^1,2^, Jing Xuan Wong^1,2^, Pui Shi Chan^1,2^, Lek Wen Tan^3^ , Roger Foo^2,3,†^, Jianming Jiang^1,2, † *^

**
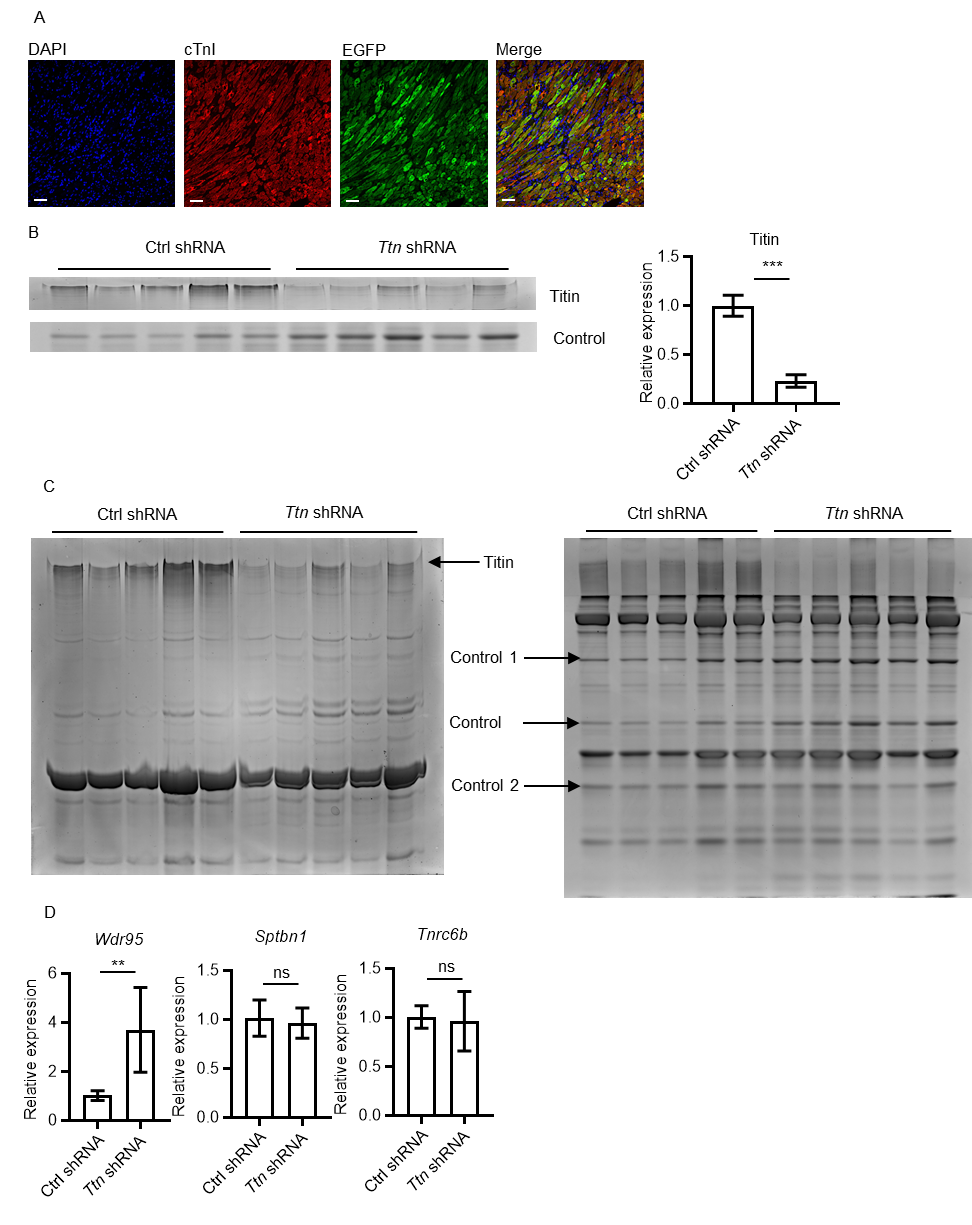
**

**Supplementary Fig. S1 *Ttn* shRNA suppresses *Ttn* protein level.**

(**A**) Paraffin sections of mouse hearts 3 weeks after AAV-cTnT-*EGFP* transduction. Samples were stained with DAPI (Blue), cTnI (Red) and *EGFP* (Green). Magnification = 20 ×, scale bar = 50 µm. (**B**) Coomassie blue staining (left) and quantitative analysis (right) of Titin protein expression in heart tissues from mice treated with control and *Ttn* shRNA respectively for three weeks. Virus dose, 0.8E+13 vg/kg. Data were normalized to average grayness of three control bands blotted, n = 5. (**C**) The full-length gel in (**B**) with identified Titin and control bands. (**D**) Quantitative real-time PCR analysis of *Ttn* shRNA off-targeted genes, n = 8.


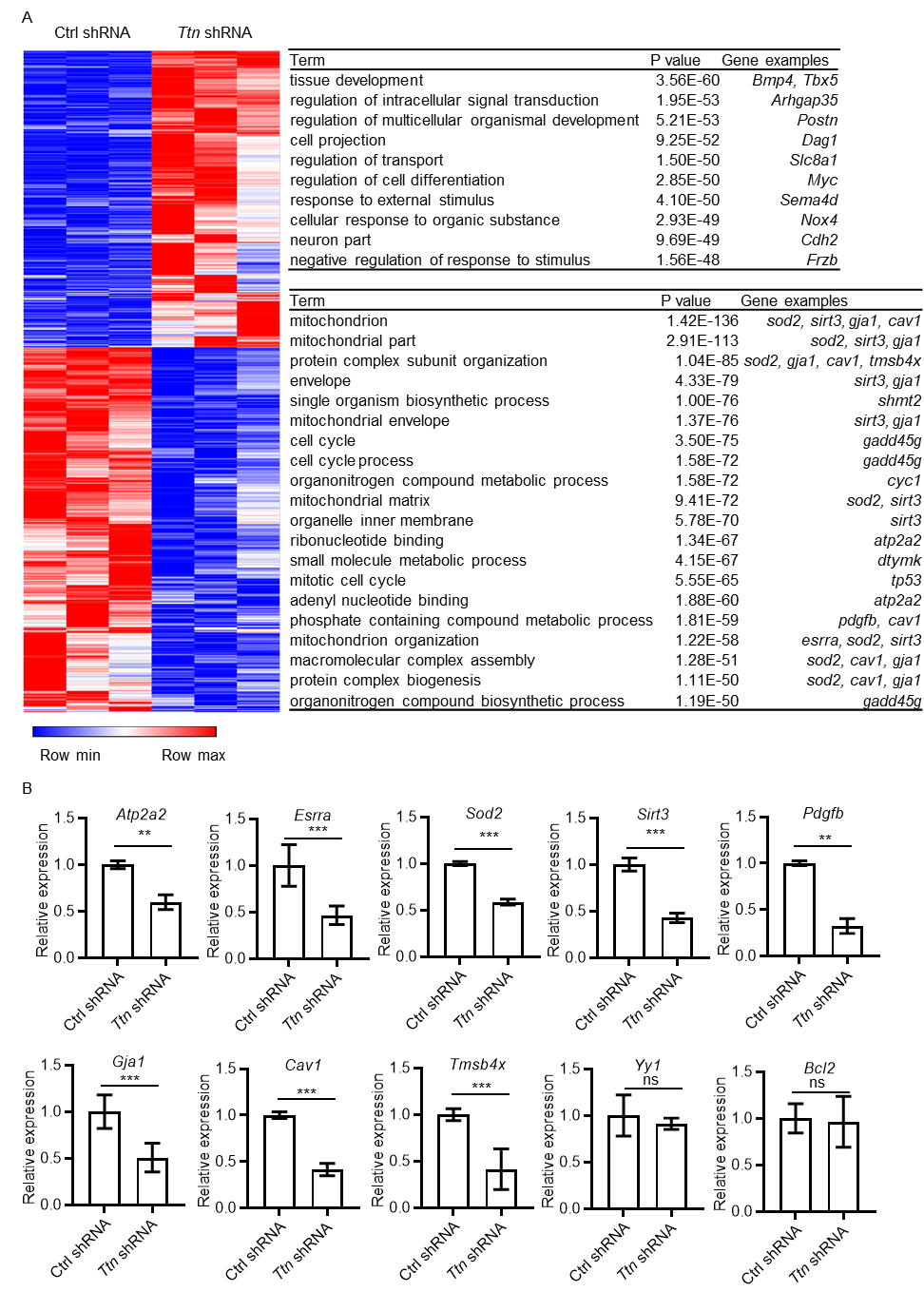


**Supplementary Fig. S2** **Genes suppressed in dilated cardiomyopathy induced by *Ttn* shRNA**.

(**A**) Heat map (left) representing color-coded expression level of 3101 differentially expressed genes in *Ttn* shRNA group compared to control shRNA group. Virus does, 0.2E+13 vg/kg. Mice were harvest four weeks after transduction, n = 3; Top 10 GO terms of up-regulated genes and top 20 GO terms of down-regulated genes, with associated P value and representative genes. (**B**) Quantitative real-time PCR analysis of selected genes in control and *Ttn* shRNA transduced mice respectively, n=3.
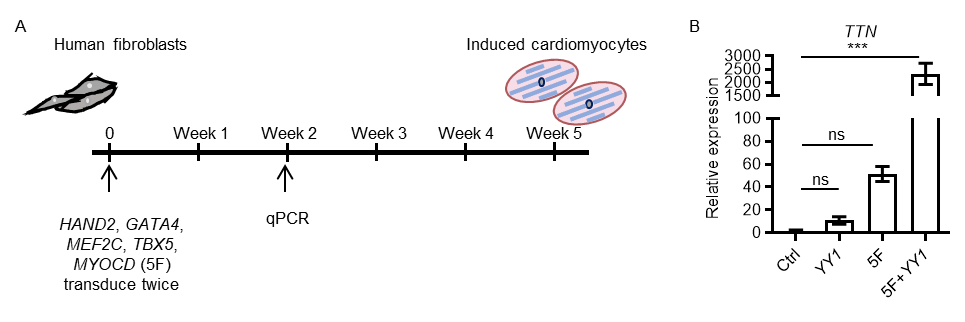


**Supplementary Fig. S3 Upregulation of *YY1* enhances *TTN* expression in human direct cardiac reprograming.**

**(A**) Experimental timeline showing time points of virus transduction and sample collection. (**B**) Quantitative real-time PCR analysis of *TTN* expression in control, *YY1*, 5F and 5F with *YY1* transduced human dermal fibroblasts. 5F includes *GATA4*, *MEF2C*, *TBX5*, *HAND2* and *MYOCD*, n = 4.


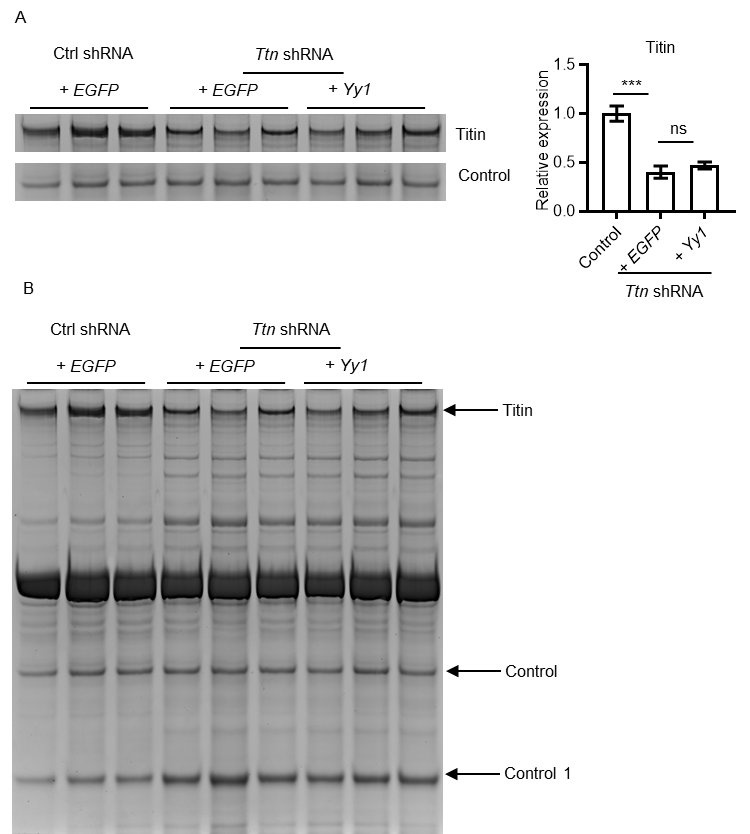


**Supplementary Fig. S4** ***Yy1* does not compensate *Ttn* protein level reduced by *Ttn* shRNA.**

(**A**) Coomassie blue staining (left) and quantitative analysis (right) of Titin protein expression in heart tissues from mice, transduced for four weeks, of control group, *Ttn* shRNA group and Yy1 treated group. Virus dose, 0.2E+13 vg/kg. Data were normalized to average grayness of two control bands blotted, n = 3. (**B**) The full-length gel of (**A**) with identified Titin and control bands.


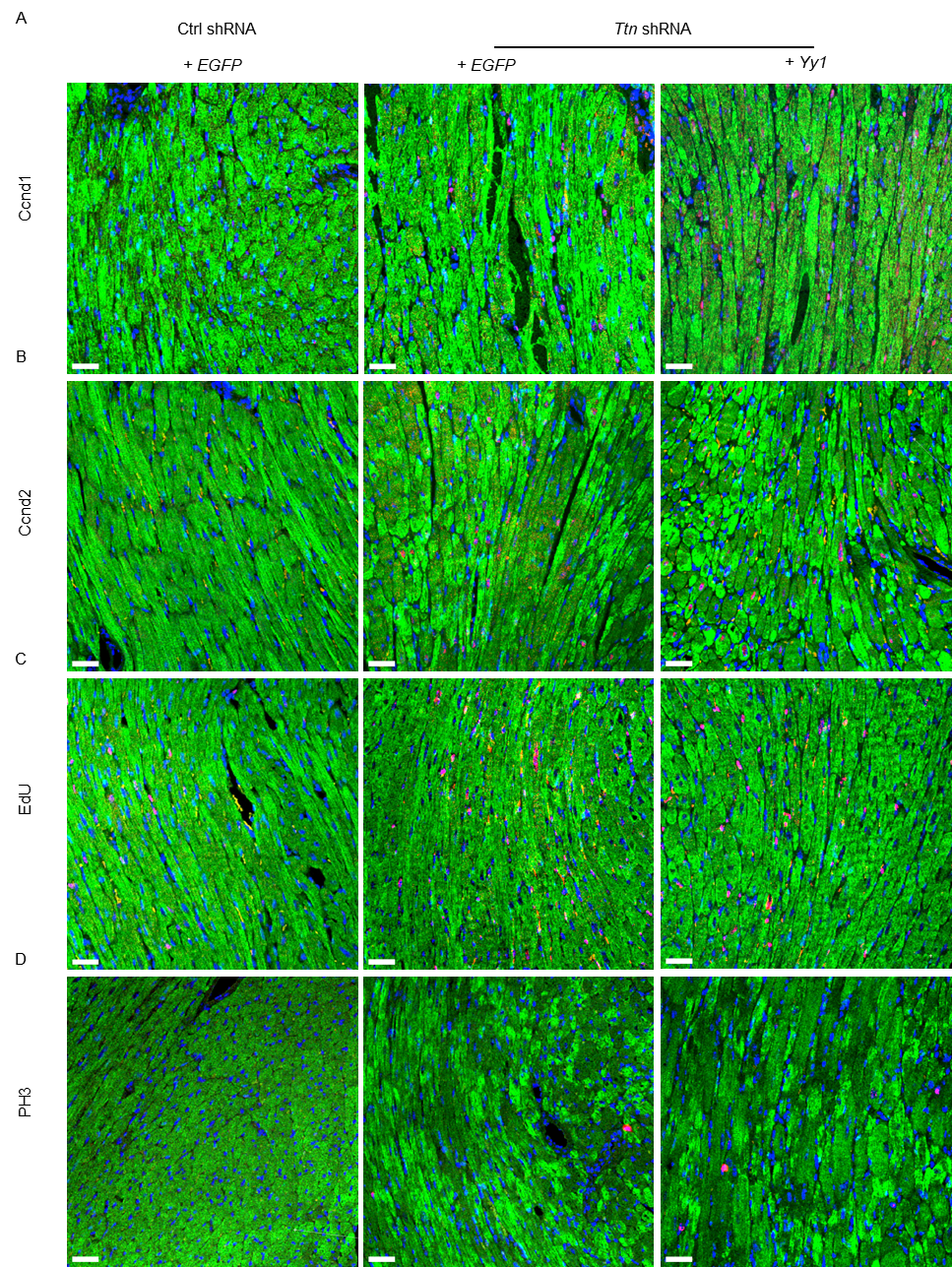


**Supplementary Fig. S5 Upregulation of *Yy1* modulates cardiac cell cycle genes.**

Paraffin sections of mice from control, *Ttn* shRNA and *Yy1* treated groups 4 weeks after virus transduction. Samples were stained or assayed with DAPI (Blue), cTnI (green) and Ccnd1, Ccnd2 and EdU (red) in (**A**), (**B**), (**C**) and (**D**). Virus dose, 0.2E+13 vg/kg. Magnification = 20 ×, scale bar = 50 µm, n=4.

**
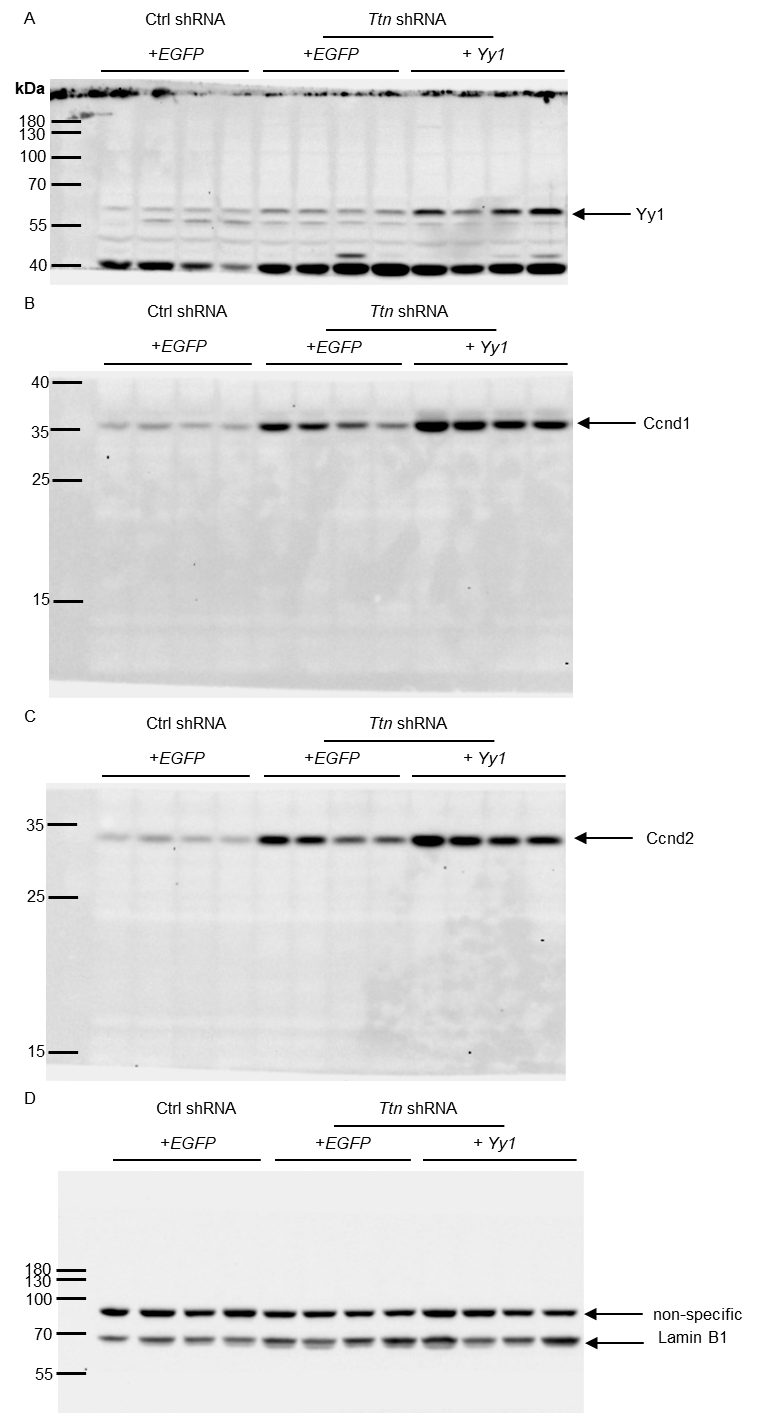
**

**Supplementary Fig. S6 Upregulation of *Yy1* promotes cell cycle gene expression**

Completed western blot of (**A**) Yy1, (**B**) Ccnd1 and (**C**) Ccnd2 protein expression level in heart tissues from mice, transduced for four weeks, of control group, *Ttn* shRNA group and Yy1 treated group. Virus dose, 0.2E+13 vg/kg. Data were normalized to the grayness of an unspecific bands blotted by Lamin B1 antibody (**D**), n = 4.

| Gene | Function |
| --- | --- |
| *Atp2a2* | ATPase Sarcoplasmic/Endoplasmic Reticulum Ca2+ Transporting 2 |
| *Esrra* | Estrogen-related receptor Alpha, transcriptional regulator |
| Sod2 | Superoxide Dismutase 2, Destroys superoxide anion radicals which are toxic |
| Sirt3 | Sirtuin 3, intracellular regulatory proteins with mono-ADP-ribosyltransferase activity |
| *Pdgfb* | Platelet Derived Growth Factor Subunit B |
| *Gja1* | Gap Junction Protein Alpha 1, intercellular channels |
| *Bcl2* | B-cell lymphoma 2, apoptosis regulator |
| *Yy1* | Yin Yang 1, transcriptional regulator |
| *Cav1* | Caveolin 1, scaffolding protein |
| *Tmsb4x* | Thymosin Beta 4 X-linked |

**Supplementary Table. S1 The summary of selected genes.**

10 gene candidates that were selected to verify their potential therapeutic function in *Ttn* shRNA transduced mice were listed with respective functions.

| **Does** | **Sex** | **Age** | **Virus** | **N** | **LVDD** | **P** | **LVWT** | **P** | **EF%** | **P** | **FS%** | **P** |
| --- | --- | --- | --- | --- | --- | --- | --- | --- | --- | --- | --- | --- |
| 2 ×  (0.2E+13) | M | 4.5  weeks | Ctrl shRNA  + *EGFP* | 15 | 3.75±0.19 | *** | 0.62±0.04 | *** | 59.32±4.06 | *** | 30.95±2.72 | *** |
|  |  |  | *Ttn* shRNA  + *EGFP* | 20 | 4.22±0.23 |  | 0.49±0.03 |  | 21.71±9.07 |  | 9.91±4.32 |  |
|  |  |  | *Ttn* shRNA  + *Yy1* | 5 | 3.91±0.15 | * | 0.52±0.04 | ns | 34.91±2.65 | ** | 16.39±1.44 | ** |
|  |  |  | *Ttn* shRNA  +*Pdgfb* | 4 | 4.10±0.11 | ns | 0.47±0.05 | ns | 27.50±4.65 | ns | 12.67±2.28 | ns |
|  |  |  | *Ttn* shRNA  + *Sod2* | 4 | 4.26±0.36 | ns | 0.45±0.05 | ns | 23.80±2.29 | ns | 10.88±1.00 | ns |
|  |  |  | *Ttn* shRNA  + *Gja1* | 4 | 4.29±0.14 | ns | 0.43±0.02 | * | 21.85±7.06 | ns | 9.94±3.48 | ns |
|  |  |  | *Ttn* shRNA  + *Bcl2* | 4 | 4.19±0.21 | ns | 0.51±0.05 | ns | 21.29±5.11 | ns | 9.62±2.48 | ns |
|  |  |  | *Ttn* shRNA  + *Atp2a2* | 5 | 4.18±0.20 | ns | 0.51±0.07 | ns | 19.02±5.00 | ns | 8.52±2.37 | ns |
|  |  |  | *Ttn* shRNA  + *Esrra* | 5 | 4.09±0.13 | ns | 0.46±0.03 | ns | 19.31±7.02 | ns | 8.68±3.35 | ns |
|  |  |  | *Ttn* shRNA  + Cav1 | 4 | 3.96±0.29 | ns | 0.54±0.02 | ns | 13.75±5.36 | ns | 6.03±2.41 | ns |
|  |  |  | *Ttn* shRNA  + Tmsb4x | 4 | 4.08±0.23 | ns | 0.51±0.03 | ns | 13.14±2.37 | ns | 5.76±1.06 | ns |
|  |  |  | *Ttn* shRNA  + *Sirt3* | 4 | 4.69±0.29 | ** | 0.45±0.01 | ns | 11.75±4.50 | ns | 5.21±2.04 | ns |

**Supplementary Table. S2 Effect of gene candidates on cardiac morphology and function of *Ttn* shRNA transduced mice.**

Potential therapeutic effect of selected genes on cardiac morphology and function of DCM mice induced by *Ttn* shRNA. Positive candidates with EF greater than *Ttn* shRNA group (*Ttn* shRNA + *EGFP*) were highlighted as yellow, otherwise as blue. P value, representing comparison to *Ttn* shRNA group, was obtained by ANOVA and Tukey’s multiple comparisons test. LVDD, left ventricular diastolic dimension; LVWT, LV wall thickness; EF, ejection fraction; FS, fractional shortening.

| **Does** | **Sex** | | **Age** | **Virus** | **N** | **LVDD** | **P** | **LVWT** | **P** | **EF%** | **P** | **FS%** | **P** |
| --- | --- | --- | --- | --- | --- | --- | --- | --- | --- | --- | --- | --- | --- |
| 2 ×  (0.2E+13) | M | 4.5  weeks | | Ctrl shRNA  +*EGFP* | 10 | 3.62±0.18 | *** | 0.62±0.04 | *** | 58.35±3.96 | *** | 30.20±2.69 | *** |
|  |  |  |  | *Ttn* shRNA  +*EGFP* | 12 | 4.20±0.34 |  | 0.49±0.05 |  | 23.17±7.23 |  | 10.56±3.43 |  |
|  |  |  |  | *Ttn* shRNA  +*Yy1* | 8 | 3.90±0.18 | * | 0.54±0.09 | ns | 31.91±5.70 | ** | 14.85±2.88 | * |
|  |  | 5.5  weeks | | Ctrl shRNA  +*EGFP* | 10 | 3.75±0.27 | *** | 0.64±0.07 | *** | 55.90±4.75 | *** | 28.69±3.03 | *** |
|  |  |  |  | *Ttn* shRNA  +*EGFP* | 9 | 4.29±0.31 |  | 0.50±0.06 |  | 17.41±4.41 |  | 7.79±2.08 |  |
|  |  |  |  | *Ttn* shRNA  +*Yy1* | 8 | 3.81±0.19 | ** | 0.56±0.09 | ns | 35.40±4.17 | *** | 16.62±2.20 | *** |
|  |  | 6.5  weeks | | Ctrl shRNA  +*EGFP* | 10 | 3.67±0.21 | *** | 0.64±0.02 | *** | 57.01±2.56 | *** | 29.31±1.62 | *** |
|  |  |  |  | *Ttn* shRNA  +*EGFP* | 5 | 4.59±0.28 |  | 0.48±0.02 |  | 12.52±2.06 |  | 5.53±0.91 |  |
|  |  |  |  | *Ttn* shRNA  +*Yy1* | 8 | 4.01±0.20 | *** | 0.54±0.05 | * | 27.13±3.59 | *** | 12.43±1.77 | *** |
|  |  | 7.5  weeks | | Ctrl shRNA  +*EGFP* | 10 | 3.71±0.29 | *** | 0.65±0.03 |  | 58.49±2.44 | *** | 30.22±1.62 | *** |
|  |  |  |  | *Ttn* shRNA  +*EGFP* | 3 | 4.89±0.25 |  | 0.46±0.01 |  | 8.15±0.79 |  | 3.58±0.36 |  |
|  |  |  |  | *Ttn* shRNA  +*Yy1* | 8 | 4.05±0.12 | *** | 0.51±0.04 | ns | 22.99±3.25 | *** | 10.39±1.57 | *** |

**Supplementary Table. S3 Effect of *Yy1* on cardiac morphology and function of *Ttn* shRNA transduced mice.**

Therapeutic effect of *Yy1* treatment in *Ttn* shRNA transduced mice with four-timepoint assessments. P value, representing comparison to *Ttn* shRNA group (*Ttn* shRNA + *EGFP*) at respective timepoint, was obtained by ANOVA and Tukey’s multiple comparisons test. LVDD, left ventricular diastolic dimension; LVWT, LV wall thickness; EF, ejection fraction; FS, fractional shortening.

| **Dose** | **Sex** | **Age** | **Virus** | **N** | **LVDD** | **P** | **LVWT** | **P** | **EF%** | **P** | **FS%** | **P** |
| --- | --- | --- | --- | --- | --- | --- | --- | --- | --- | --- | --- | --- |
| 0.2E+13 | M | 5.5  weeks | *EGFP* | 5 | 3.73±0.16 |  | 0.60±0.03 |  | 54.65±1.55 |  | 27.86±1.00 |  |
|  |  |  | *Yy1* | 5 | 3.69±0.08 | ns | 0.62±0.05 | ns | 53.66±2.20 | ns | 27.13±1.36 | ns |
|  |  | 10.5  weeks | *EGFP* | 5 | 3.96±0.12 |  | 0.62±0.04 |  | 54.25±1.56 |  | 27.66±1.08 |  |
|  |  |  | *Yy1* | 5 | 3.85±0.11 | ns | 0.64±0.03 | ns | 53.43±1.36 | ns | 27.06±0.83 | ns |
|  |  | 21.5  weeks | *EGFP* | 5 | 4.03±0.16 |  | 0.62±0.03 |  | 52.36±2.28 |  | 26.51±1.47 |  |
|  |  |  | *Yy1* | 5 | 4.09±0.16 | ns | 0.64±0.03 | ns | 52.78±2.28 | ns | 26.81±1.46 | ns |

**Supplementary Table. S4** **Effect of *Yy1* on cardiac morphology and function of wildtype mice.**

| **Dose** | **Sex** | **Age** | **Virus** | **N** | **LVDD** | **P** | **LVWT** | **P** | **EF%** | **P** | **FS%** | **P** |
| --- | --- | --- | --- | --- | --- | --- | --- | --- | --- | --- | --- | --- |
| 2 ×  (0.2E+13) | M | 4.5  weeks | Ctrl shRNA + *EGFP* | 9 | 3.66±0.14 | ** | 0.62±0.02 | * | 58.67±3.07 | *** | 30.44±2.06 | *** |
|  |  |  | *Ttn* shRNA + *EGFP* | 10 | 4.14±0.36 |  | 0.52±0.08 |  | 23.37±8.34 |  | 10.67±4.02 |  |
|  |  |  | *Ttn* shRNA + *Ccnd1* | 5 | 3.79±0.14 | ns | 0.64±0.06 | * | 34.74±3.23 | * | 16.25±1.71 | * |
|  |  |  | *Ttn* shRNA + *Ccnd2* | 5 | 3.74±0.12 | * | 0.60±0.04 | ns | 34.65±5.24 | * | 16.22±2.79 | * |
|  |  | 5.5  weeks | Ctrl shRNA + *EGFP* | 9 | 3.94±0.13 | *** | 0.62±0.03 | *** | 55.47±3.06 | *** | 28.46±1.97 | *** |
|  |  |  | *Ttn* shRNA + *EGFP* | 6 | 4.41±0.19 |  | 0.47±0.04 |  | 12.22±4.87 |  | 5.40±2.21 |  |
|  |  |  | *Ttn* shRNA + *Ccnd1* | 5 | 3.95±0.31 | ** | 0.57±0.06 | ** | 30.44±8.46 | *** | 14.16±4.36 | *** |
|  |  |  | *Ttn* shRNA + *Ccnd2* | 5 | 4.26±0.18 | ns | 0.54±0.03 | * | 27.13±5.93 | *** | 12.52±2.99 | ** |

P value, representing comparisons to *EGFP* transduced mice at respective age, was obtained by unpaired T-test. LVDD, left ventricular diastolic dimension; LVWT, LV wall thickness; EF, ejection fraction; FS, fractional shortening.

**Supplementary Table. S5** **Effect of *Ccnd1* and *Ccnd2* on cardiac morphology and function of *Ttn* shRNA transduced mice.**

P value were obtained by ANOVA and Tukey’s multiple comparisons test, reflecting comparisons of cardiac dimensions or function to *Ttn* shRNA group (*Ttn* shRNA + *EGFP*). LVDD, left ventricular diastolic dimension; LVWT, LV wall thickness; EF, ejection fraction; FS, fractional shortening.
